# The association between stereoscopic vision and cognitive function on community-dwelling older adults: a cross-sectional study

**DOI:** 10.1186/s12877-025-06014-4

**Published:** 2025-05-20

**Authors:** Yeon Jung Park, Hyun Jin Cho, Kiyoung Kim, Chang Won Won, Miji Kim

**Affiliations:** 1 College of Medicine, Kyung Hee University, Seoul, Korea; 2 Department of Health Sciences and Technology, College of Medicine, Kyung Hee University, Seoul, Korea; 3 Department of Ophthalmology, Kyung Hee University Hospital, Kyung Hee University, Seoul, Korea; 4 Elderly Frailty Research Center, Department of Family Medicine, College of Medicine, Kyung Hee University, Seoul, Korea

**Keywords:** Cognitive function, Cognitive impairment, Stereopsis, Stereoscopic vision, Older adults

## Abstract

**Background:**

Visual impairment is associated with cognitive dysfunction in older adults; however, a relationship between stereopsis and cognitive function remains unclear. This study aimed to investigate the association between stereopsis and cognitive function in community-dwelling older adults.

**Methods:**

A cross-sectional analysis was conducted with data of 1,228 participants aged 74–88 years from the Korean Frailty and Aging Cohort Study. Cognitive function was assessed by the Korean version of the Consortium to Establish a Registry for Alzheimer’s Disease Assessment Packet (CERAD-K). Stereoscopic vision was measured using the Titmus Fly test and recorded in 3 categories according to the angle of stereopsis. Multiple regression analyses were used to investigate the association between stereopsis and cognitive function.

**Results:**

Total 565 men and 663 women were included. Stereopsis was associated with education, residence, and visual acuity (*p* <.05). Participants with poor and moderate stereopsis were more likely to have cognitive dysfunction than participants with good stereopsis. Poor stereopsis was negatively associated with cognitive function scores in word list memory, word list recall, Trail Making Test-A errors, Trail Making Test-A response time and frontal assessment battery (*p* <.05). Compared to good stereopsis group, moderate (odds ratio [OR] = 1.60; 95% confidence interval [CI] = 1.08–2.36) and poor (OR = 1.85; 95% CI = 1.24–2.76) stereopsis groups had a higher risk of cognitive impairment even after controlling for several covariates.

**Conclusions:**

Poor stereopsis was associated with cognitive dysfunction and had increased odds for cognitive impairment. Our findings suggest a cross-sectional association between stereopsis and cognitive dysfunction in community-dwelling older adults.

## Background

Aging is a complex biological process associated with a gradual physiological decline and increased vulnerability to various health conditions [1–3]. As life expectancy increases globally, the prevalence of age-related disorders, including sensory, cognitive, and functional impairments, is also increasing. Common health challenges in older adults include sarcopenia, frailty, visual and hearing impairments, and cognitive decline, all of which contribute to a reduced quality of life and increased care needs [1–3]. Notably, cognitive aging involves both structural and functional changes in the brain, such as reduced synaptic plasticity, cortical thinning, and neurotransmitter deficits, which can impair memory, attention, and executive functions [1, 4]. These changes often interact with other age-related sensory impairments, emphasizing the importance of investigating multisensory influences on cognition in older populations.

The prevalence of cognitive impairment, characterized by deficits in one or more cognitive functions, increases significantly with age [4]. Cognitive impairment can be classified as mild cognitive impairment (MCI) or dementia based on severity [1, 5]. Cognitive impairment causes negative health outcomes, such as falls, poor quality of life, limitations in the activities of daily living, instrumental activities of daily living, hospitalization, dementia, and mortality [2, 3]. Individuals with MCI face challenges in daily life due to cognitive decline and emotional changes, which obstruct socialization [6, 7]. Therefore, identifying risk factors for cognitive impairment is necessary to help older adults successfully perform daily activities and tasks.

Well-known risk factors associated with cognitive impairment include a low educational level, poor physical ability, social activity, and sensory impairment [8, 9]. A systematic review also reported that visual impairment was associated with cognitive impairment and dementia in older adults [10]. Basic visual functions such as near and distance visual acuity, contrast sensitivity, and stereopsis, are critical for daily activities and for maintaining cognitive engagement [11]. However, most studies on visual function and cognition have focused on visual acuity [12, 13], with only a few studies addressing contrast sensitivity [14]. Among these, the focus on stereopsis has been limited, despite its unique and important role in higher-order visual processing. Stereopsis refers to the ability to perceive the depth and distance of an object based on its binocularity [15]. It has several functions in the human vision system: first, to identify different spatial relationships in three-dimensional vision; second, to assess awareness of the distance between objects and the observer; and third, to help perform cognitive tasks, including visual memory and visual attention [16, 17]. Deficits in depth perception may lead to substantial difficulties in everyday activities [18, 19].

However, most studies on visual function and cognition have focused on visual acuity [12, 13], with only a few studies addressing contrast sensitivity [14]. Additionally, few studies have examined the relationship between stereopsis and cognitive function [20–22]. The two studies that did address this issue primarily focused on patients with neurodegenerative diseases [20, 21], whereas the other was limited to a highly educated population [22].

Therefore, this study used a comprehensive neuropsychological battery test to explore the relationship between stereopsis and cognitive function in community-dwelling older adults in Korea. It also accounted for various confounding factors, including sociodemographic, lifestyle, health-related, and sensory functional variables.

## Methods

### Study participants

This cross-sectional study used data collected from the Korean Frailty and Aging Cohort Study (KFACS). The KFACS is a nationwide, multicenter, longitudinal study that began with a baseline survey in 2016–2017. A total of 3014 community-dwelling older adults were recruited in urban and rural areas at 10 centers at the baseline with follow-up surveys conducted every 2 years. Each center recruited participants using quota sampling stratified by age (70–74, 75–79, and 80–84 years, with a ratio of 6:5:4, respectively) and sex (male and female, 1:1) [23]. The baseline survey was conducted from 2016 to 2017 (wave 1). Among them, 2,428 participants were followed up in the wave 3 (2020–2021) survey, while 3 withdrew from the study after enrollment, and 583 participants were lost during follow-up (128 deaths, 13 institutionalizations, 7 hospitalizations, 166 unreachable, 258 simple rejections, 3 moved away from the study area, and 8 participants for other reasons). For those who were followed up, participants who underwent just telephone survey (*n* = 422) or proxy interviews (*n* = 52) instead of research center visiting were excluded. Participants without information on the following variables that could be considered confounders were also excluded from the analysis: Mini-Mental State Examination (MMSE) scores < 10 (*n* = 9), stereopsis (*n* = 4), cognitive function (*n* = 33), other demographic and health-related confounders (*n* = 465), and the inability to grasp fly wings (*n* = 197). However, some of the participants who were categorized as ‘unable to grasp fly wings’ were also excluded for other reasons, including missing data on cognitive function or missing data on covariates; thus, the exclusion categories were not mutually exclusive.

**Fig. 1:**
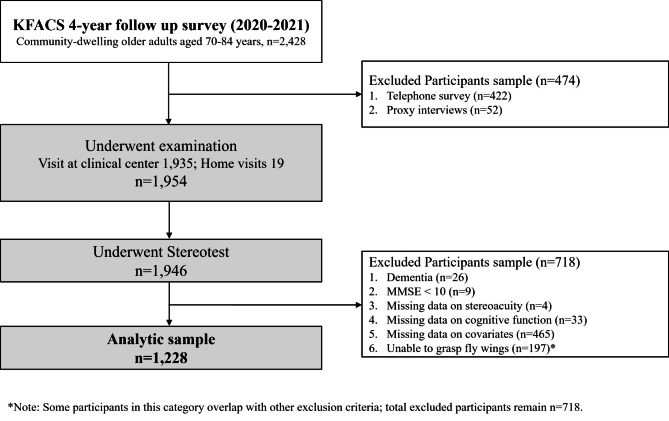
Flowchart of participant selection at baseline. KFACS, Korean Frailty and Aging Cohort Study MMSE, Mini-Mental State Examination

Finally, 1,228 patients (565 males and 663 females) were included in this analysis. The participant selection flowchart for this study is shown in Fig. 1. This study used wave 3 data because stereoacuity test was conducted from wave 3. The KFACS protocol was approved by the Clinical Research Ethics Committee of Kyung Hee University Hospital (Institutional Review Board number: 2015-12-103). This study was exempted from further review by the Clinical Research Ethics Committee of Kyung Hee University Medical Center (IRB No. 2025-03-033) and conducted in accordance with the Declaration of Helsinki. All participants provided written informed consent.

### Measurements

#### Assessment of cognitive function

Cognitive function was measured using a neuropsychological battery test, the Korean version of Consortium to Establish a Registry for Alzheimer’s Disease (CERAD-K), which include the Korean version of the MMSE (MMSE-KC). Verbal memory was measured using three categories: word list memory, recall, and recognition tests. The processing speed was measured using the Trail Making Test A (TMT-A). Attention was measured using the digit span (forward and backward) test. Executive function was measured using frontal assessment battery (FAB) scores.

The word list memory test was used to evaluate the ability to register new information by recalling 10 different nouns three times. The word list recall test was used to test delayed recall memory. Participants were shown 10 words and asked to recall as many words as possible after a few minutes. The word list recognition test was used to test recognition memory by classifying 10 previously seen words and 10 new words. The digit span forward test is regarded as more related to attention, whereas the digit span backward test is used to evaluate working memory. The digit span test numbers were composed of three to nine digits in the forward test and two to eight digits in the backward test. The final score was calculated as the sum of the forward and backward digit span scores. The TMT-A assesses sequencing, processing speed, and visuospatial ability. Participants were asked to connect 25 dots as quickly as possible, and the connected number of dots, number of errors, and time taken were measured. The FAB consists of six subtests: conceptualization, mental flexibility, motor programming, sensitivity to interference, inhibitory control, and environmental autonomy. Each subtest had a perfect score of three points, with a total score of 18 points.

Cognitive impairment was defined as having less than 1.5 standard deviation (SD) of Korean norm according to sex, age, education in any one of the cognitive function tests including TMT-A, FAB, digit span backward, and word list recall test [24].

The MMSE-KC is a global cognitive function screening test consisting of five domains: orientation (10 points), memory (6 points), attention (5 points), language ability (6 points), and comprehension and judgment (3 points) [25].

The validity of these questionnaires is well-established. For example, the MMSE-KC, part of the CERAD-K Assessment Packet, is a modified version adapted for Korea’s high illiteracy rate among older adults [25]. This is distinct from the K-MMSE (or K-MMSE-2) [26], which is equivalent to the MMSE (or MMSE-2), and has been used in other research [27, 28].

#### Visual function measurement

Distance and near visual acuity were measured binocularly. Distance visual acuity was measured using Han’s vision chart at a distance of 4 m and near visual acuity was measured using a near point card at a distance of 40 cm. The participants were asked to wear habitual optical correction, if any. Visual acuity scores were converted into logarithm of the minimum angle of resolution (logMAR) notation [29].

Stereopsis was measured using the Titmus Fly test at a 40 cm distance with both eyes open [30, 31]. Polaroid glasses were worn for near-refractive correction, if any. The patients were first asked to grasp the wings of the fly. If the patient correctly grasped the wings, an additional test was performed to measure the angle of stereopsis by observing nine sets of four circles and three rows of five animals [31]. The angle of stereopsis was recorded according to the grade chart by choosing the last circle that seemed to be closer to the participant. The results of the stereopsis test were classified into three categories based on the angle of stereopsis: good (40–60 arcsec), moderate (80–200 arcsec), and poor (> 200 arcsec) [32].

#### Other measurement

Information on the research participants, including age, sex, education, marital status, social security recipient, and other sociodemographic and lifestyle information, was obtained through a trained investigator using a structured questionnaire. The residential areas of the participants were sorted into three categories: urban, suburban, and rural. The body mass index (BMI) was calculated as body weight divided by height squared. Lifestyle factors were examined, including alcohol at least 23 times/week and current smoking status. Energy expenditure estimates (kcal/week) for physical activity levels were measured using the International Physical Activity Questionnaire in a population-based Korean survey of older adults [23]. Low physical activity was defined as the lowest 20% of the sex-specific total energy consumed, which was < 494.65 kcal for males and < 283.50 kcal for females per week [23]. Medical conditions, such as hypertension, dyslipidemia, myocardial infarction, congestive heart failure, angina pectoris, diabetes mellitus, and chronic kidney disease, were recorded based on self-reported physician diagnoses. Depressive symptoms were evaluated using the Korean version of the Short Form of the Geriatric Depression Scale, with a score ≥ 6 indicating the presence of depressive symptoms [33]. Ocular pathologies including cataracts, glaucoma, diabetic retinopathy, macular degeneration, and blindness, were assessed through medical history-taking, while visual functions, including visual acuity and stereopsis, were measured by a trained investigator. Patients with at least one of the previously described pathologies were considered to have visual pathology.

Hearing impairment was defined as a pure-tone average (average hearing threshold at 0.5, 1.0, 2.0, and 4.0 kHz) of 40 dB or more in the better-hearing ear, in accordance with previous studies [34].

### Statistical analysis

In this study, differences in characteristics across stereopsis levels were analyzed using the chi-square test for categorical variables and analysis of variance for continuous variables. Continuous variables are expressed as means and standard deviations, and categorical variables are expressed as numbers and percentages. The association between stereopsis and cognitive function was investigated using linear regression analyses adjusted for multiple confounders, and the association between stereopsis and cognitive impairment was investigated using logistic regression analysis adjusted for the same confounders. We included the confounders identified between cognitive function and stereopsis from previous studies [12, 35, 36]. To account for confounders, all models were adjusted as follows: Model 1 was unadjusted; Model 2 was adjusted for age, sex, education, marital status, residence, and social security recipient; Model 3 was further adjusted for smoking status, alcohol intake, low physical activity, and BMI; Model 4 was further adjusted for medical condition and eye diseases (cataracts, glaucoma, diabetic retinopathy, macular degeneration, and blindness); Model 5 was further adjusted for near visual acuity; and Model 6 was further adjusted for hearing impairment. The measures employed by the associations (odds ratio [OR] or beta [β]) and their respective confidence intervals (CIs) were reported. All analyses were performed using IBM SPSS Statistics (version 27.0; IBM Corp., Armonk, NY). A two-sided *p* value of ≤ 0.05 was considered statistically significant.

## Results

### Baseline characteristics of the study population

The demographic and clinical characteristics of the study population according to stereopsis level are shown in Table 1. According to the level of stereopsis, 27.4%, 38.7%, and 34.0% of the total participants were classified into good, moderate, and poor groups, respectively. Participants with good stereopsis were more likely to have higher education, to live in urban areas, and exhibit better distance and near visual acuity compared to those with poor and moderate stereopsis. Regarding sociodemographic factors, sex, marital status, and social security were not significantly associated with stereopsis (*p* >.05). BMI and lifestyle factors such as smoking status, alcohol intake, low physical activity, and medical conditions were not significantly associated with stereopsis (*p* >.05). Eye diseases and hearing impairment were not significantly associated with stereopsis (*p* >.05).

**Table 1:** Characteristics of the study population by stereopsis

Variable	Overall (*n* = 1228)	Stereopsis			*p* -value
		Good 40–60 arcsec	Moderate 80–200 arcsec	Poor > 200 arcsec	
***Socio-demographic factors***					
Age (years)	80.0 ± 3.6	79.6 ± 3.6	80.4 ± 3.6	79.9 ± 3.6	**0.007**
Women, %	663 (54.0)	178 (53.0)	255 (53.7)	230 (55.2)	0.825
BMI (kg/m^2^)	24.7 ± 12.2	24.5 ± 3.0	25.3 ± 19.3	24.3 ± 3.0	0.452
Years of education	9.1 ± 6.6	9.8 ± 4.6	9.1 ± 6.5	8.5 ± 8.0	**0.025**
Marital status (with partner) %	777 (63.3)	220 (65.5)	293 (61.7)	264 (63.3)	0.544
Residence, %					**0.028**
Urban	308 (25.1)	69 (20.5)	122 (25.7)	117 (28.1)	
Suburban	563 (45.8)	176 (52.4)	217 (45.7)	170 (40.8)	
Rural	357 (29.1)	91 (27.1)	136 (28.6)	130 (31.2)	
Social security recipient, %	76 (6.2)	16 (4.8)	35 (7.4)	25 (6.0)	0.310
***Lifestyle factors***					
Current smoker, %	48 (3.9)	12 (3.6)	18 (3.8)	18 (4.3)	0.859
Alcohol (≥ 2–3 time/week), %	171 (13.9)	49 (14.6)	66 (13.9)	56 (13.4)	0.902
Low physical activity, %	113 (9.2)	30 (8.9)	41 (8.6)	42 (10.1)	0.744
***Medical condition***					
Hypertension, %	757 (61.6)	195 (58.0)	310 (65.3)	252 (60.4)	0.093
Dyslipidemia, %	563 (45.8)	162 (48.2)	219 (46.1)	182 (43.6)	0.453
Myocardial Infarction, %	34 (2.8)	4 (1.2)	16 (3.4)	14 (3.4)	0.118
Congestive heart failure, %	24 (2.0)	10 (3.0)	7 (1.5)	7 (1.7)	0.277
Angina pectoris, %	100 (8.1)	34 (10.1)	42 (8.8)	24 (5.8)	0.073
Diabetes mellitus, %	307 (25.0)	72 (21.4)	130 (27.4)	105 (25.2)	0.156
Chronic kidney disease, %	23 (1.9)	7 (2.1)	9 (1.9)	7 (1.7)	0.920
Depressive symptoms (SGDS-K score), %	296 (24.1)	83 (24.7)	117 (24.6)	96 (23.0)	0.817
***Sensory functions***					
Eye Disease, %	260 (21.2)	69 (20.5)	94 (19.8)	97 (23.3)	0.424
Near Visual Acuity (logMAR)	0.45 ± 0.17	0.36 ± 0.13	0.45 ± 0.15	0.52 ± 0.18	**< 0.001**
Distance Visual Acuity (logMAR)	0.11 ± 0.15	0.06 ± 0.11	0.11 ± 0.14	0.15 ± 0.16	**< 0.001**
Hearing Impairment, %	891 (72.6)	246 (73.2)	350 (73.7)	295 (70.7)	0.587

Note: Values are presented as mean ± standard deviation or as *N* (%). BMI, body mass index. *P*-values were determined by the Chi-square test and the One-Way Analysis of Variance (ANOVA) test

Table 2 presents the cognitive function of the study population according to stereopsis level. Participants with poor stereopsis were more likely to have lower cognitive function scores and cognitive impairment (*p* <.001). Most cognitive functions showed statistically significant association with stereopsis; MMSE score, word list memory, digit span forward, digit span backward, digit span total, TMT-A error, TMT-A response time, FAB score, and cognitive impairment were all statistically significant (*p* <.001). The word list recall (*p* =.008) and the TMT-A correct response (*p* =.037) were also significantly lower in the poor stereopsis group. Only the word list recognition was not associated with stereopsis(*p* =.437).

**Table 2:** Cognitive function of the study population by stereopsis

Variable	Overall (*n* = 1228)	Stereopsis			*p* -value
		Good 40–60 arcsec	Moderate 80–200 arcsec	Poor > 200 arcsec	
***Global cognitive function***					
Mini-mental state examination, score	25.9 ± 3.2	26.5 ± 2.7	25.8 ± 3.2	25.5 ± 3.5	**< 0.001**
***Verbal memory***					
Word list memory, score	17.9 ± 4.4	18.8 ± 4.0	17.5 ± 4.4	17.6 ± 4.6	**< 0.001**
Word list recall, score	5.8 ± 2.2	6.1 ± 2.2	5.7 ± 2.1	5.8 ± 2.2	**0.008**
Word list recognition, score	8.8 ± 3.0	8.8 ± 1.6	8.9 ± 4.2	8.6 ± 2.0	0.437
***Attention***					
Digit span forward, score	5.9 ± 2.5	6.4 ± 2.5	5.9 ± 2.5	5.7 ± 2.4	**< 0.001**
Digit span backward, score	4.0 ± 1.7	4.3 ± 1.6	4.0 ± 1.8	3.8 ± 1.7	**< 0.001**
Digit span total, score	9.9 ± 3.7	10.7 ± 3.6	9.9 ± 3.8	9.4 ± 3.5	**< 0.001**
***Processing speed***					
Trail making test-A, score	24.8 ± 1.5	25.0 ± 0.0	24.8 ± 1.7	24.8 ± 1.7	**0.037**
Trail making test-A, score	0.5 ± 0.9	0.3 ± 0.6	0.5 ± 1.0	0.6 ± 1.0	**< 0.001**
Trail making test-A, s	80.7 ± 62.4	62.6 ± 33.3	82.9 ± 68.1	92.6 ± 69.9	**< 0.001**
***Executive function***					
Frontal assessment battery, score	13.8 ± 3.1	14.6 ± 2.5	13.6 ± 3.3	13.3 ± 3.3	**< 0.001**
Cognitive Impairment, %	273 (22.2)	50 (14.9)	110 (23.2)	113 (27.1)	**< 0.001**

Note: Values are presented as mean ± standard deviation. *P-*values were determined by the One-Way Analysis of Variance (ANOVA) test

### Multivariable regression analysis of stereopsis and cognitive function

Table 3 presents the association between stereopsis and cognitive function using multivariate linear regression analysis. After full adjustments with confounders, the moderate stereopsis group was associated with word list memory (β=−0.09 95% CI = − 1.41, − 0.27), word list recall (β=−0.08, 95% CI = − 0.63, − 0.05), TMT-A error (β = 0.07, 95% CI = 0.00, 0.26), TMT-A response time (β = 0.10, 95% CI = 5.49, 20.78), and FAB (β=−0.10, 95% CI = − 1.04, − 0.26). The poor stereopsis group was also associated with all cognitive function factors, except word list recall, word list recognition and TMT-A correct responses (Model 5). MMSE score (β=−0.08, 95% CI = − 0.98, − 0.09), word list memory (β=−0.09, 95% CI = − 1.40, − 0.18), digit span forward (β=−0.09, 95% CI = − 0.81, − 0.12), digit span backward (β=−0.07, 95% CI = − 0.50, − 0.01), digit span total (β=−0.10, 95% CI = − 1.31, − 0.31), FAB score (β=−0.13, 95% CI = − 1.27, − 0.43) showed a negative relationship with stereopsis.

**Table 3:** Linear regression analyses to determine associations between stereopsis and cognitive functions

Cognitive functions	Stereopsis	Model 1	Model 2	Model 3	Model 4	Model 5
		*β* (95% CI)				
MMSE	Good	*Ref.*	*Ref.*	*Ref.*	*Ref.*	*Ref.*
	Moderate	**-0.10 (-1.09**, **-0.20)**	-0.06 (-0.80, 0.02)	-0.06 (-0.78, 0.03)	-0.06 (-0.80, 0.01)	-0.05 (-0.71, 0.12)
	Poor	**-0.14 (-1.41**, **-0.50)**	**-0.10 (-1.12**, **-0.28)**	**-0.10 (-1.12**, **-0.28)**	**-0.11 (-1.15**, **-0.32)**	**-0.08 (-0.98**, **-0.09)**
Word list memory	Good	*Ref.*	*Ref.*	*Ref.*	*Ref.*	*Ref.*
	Moderate	**-0.14 (-1.85**, **-0.62)**	**-0.10 (-1.43**, **-0.31)**	**-0.10 (-1.42**, **-0.30)**	**-0.10 (-1.42**, **-0.30)**	**-0.09 (-1.41**, **-0.27)**
	Poor	**-0.12 (-1.76**, **-0.49)**	**-0.09 (-1.40**, **-0.25)**	**-0.09 (-1.39**, **-0.24)**	**-0.09 (-1.40**, **-0.25)**	**-0.09 (-1.40**, **-0.18)**
Word list recall	Good	*Ref.*	*Ref.*	*Ref.*	*Ref.*	*Ref.*
	Moderate	**-0.11 (-0.76**, **-0.16)**	**-0.07 (-0.61**, **-0.04)**	**-0.07 (-0.61**, **-0.04)**	**-0.07 (-0.61**, **-0.04)**	**-0.08 (-0.63**, **-0.05)**
	Poor	**-0.08 (-0.67**, **-0.06)**	-0.06 (-0.55, 0.03)	-0.05 (-0.54, 0.04)	-0.06 (-0.56, 0.03)	-0.06 (-0.60, 0.02)
Word list recognition	Good	*Ref.*	*Ref.*	*Ref.*	*Ref.*	*Ref.*
	Moderate	0.01 (-0.35, 0.48)	0.02 (-0.29, 0.53)	0.02 (-0.29, 0.54)	0.02 (-0.33, 0.51)	0.01 (-0.35, 0.48)
	Poor	-0.03 (-0.61, 0.24)	-0.02 (-0.57, 0.27)	-0.02 (-0.57, 0.28)	-0.03 (-0.61, 0.25)	-0.04 (-0.70, 0.22)
Digit span forward	Good	*Ref.*	*Ref.*	*Ref.*	*Ref.*	*Ref.*
	Moderate	**-0.10 (-0.85**, **-0.16)**	**-0.06 (-0.63**, **-0.01)**	**-0.06 (-0.63**, **0.00)**	**-0.06 (-0.63**, **0.00)**	-0.06 (-0.62, 0.02)
	Poor	**-0.14 (-1.06**, **-0.35)**	**-0.09 (-0.80**, **-0.15)**	**-0.09 (-0.79**, **-0.15)**	**-0.10 (-0.82**, **-0.17)**	**-0.09 (-0.81**, **-0.12)**
Digit span backward	Good	*Ref.*	*Ref.*	*Ref.*	*Ref.*	*Ref.*
	Moderate	**-0.08 (-0.53**, **-0.05)**	-0.04 (-0.38, 0.07)	-0.04 (-0.37, 0.07)	-0.04 (-0.35, 0.09)	-0.02 (-0.29, 0.17)
	Poor	**-0.15 (-0.79**, **-0.30)**	**-0.11 (-0.62**, **-0.16)**	**-0.11 (-0.62**, **-0.16)**	**-0.11 (-0.62**, **-0.16)**	**-0.07 (-0.50**, **-0.01)**
Digit span total	Good	*Ref.*	*Ref.*	*Ref.*	*Ref.*	*Ref.*
	Moderate	**-0.11 (-1.33**, **-0.31)**	**-0.07 (-0.96**, **-0.05)**	**-0.07 (-0.95**, **-0.04)**	**-0.06 (-0.93**, **-0.02)**	-0.05 (-0.86, 0.07)
	Poor	**-0.17 (-1.86**, **-0.81)**	**-0.12 (-1.43**, **-0.49)**	**-0.12 (-1.43**, **-0.49)**	**-0.13 (-1.45**, **-0.51)**	**-0.10 (-1.31**, **-0.31)**
TMT-A correct response	Good	*Ref.*	*Ref.*	*Ref.*	*Ref.*	*Ref.*
	Moderate	**-0.08 (-0.46**, **-0.05)**	**-0.07 (-0.42**, **-0.01)**	**-0.07 (-0.42**, **-0.01)**	**-0.07 (-0.42**, **-0.02)**	-0.05 (-0.36, 0.05)
	Poor	**-0.72 (-0.43**, **-0.01)**	-0.06 (-0.38, 0.04)	-0.05 (-0.38, 0.04)	-0.06 (-0.38, 0.04)	-0.02 (-0.27, 0.18)
TMT-A error	Good	*Ref.*	*Ref.*	*Ref.*	*Ref.*	*Ref.*
	Moderate	**0.11 (0.08**, **0.34)**	**0.09 (0.05**, **0.30)**	**0.09 (0.05**, **0.30)**	**0.09 (0.06**, **0.31)**	**0.07 (0.00**, **0.26)**
	Poor	**0.16 (0.18**, **0.44)**	**0.14 (0.14**, **0.40)**	**0.14 (0.14**, **0.40)**	**0.14 (0.15**, **0.41)**	**0.09 (0.05**, **0.32)**
TMT-A response time	Good	*Ref.*	*Ref.*	*Ref.*	*Ref.*	*Ref.*
	Moderate	**0.16 (11.77**, **28.91)**	**0.12 (8.13**, **23.29)**	**0.12 (7.99**, **23.03)**	**0.12 (8.35**, **23.41)**	**0.10 (5.49**, **20.78)**
	Poor	**0.23 (21.22**, **38.85)**	**0.19 (17.26**, **32.85)**	**0.19 (17.11**, **32.58)**	**0.19 (17.53**, **33.04)**	**0.15 (11.84**, **28.30)**
Frontal assessment battery	Good	*Ref.*	*Ref.*	*Ref.*	*Ref.*	*Ref.*
	Moderate	**-0.16 (-1.43**, **-0.56)**	**-0.12 (-1.12**, **-0.36)**	**-0.11 (-1.12**, **-0.35)**	**-0.11 (-1.10**, **-0.33)**	**-0.10 (-1.04**, **-0.26)**
	Poor	**-0.20 (-1.73**, **-0.84)**	**-0.15 (-1.38**, **-0.59)**	**-0.15 (-1.36**, **-0.58)**	**-0.15 (-1.37**, **-0.58)**	**-0.13 (-1.27**, **-0.43)**

Note: β, beta coefficient; CI, confidence interval. Model 1: Unadjusted; Model 2: Adjusted for age, sex, years of education, marital status, residence, and social security recipient; Model 3: Further adjusted for current smoking, alcohol intake, low physical activity, and body mass index; Model 4: Further adjusted for medical condition, and eye diseases (cataract, glaucoma, diabetic retinopathy, macular degeneration, and blindness); Model 5: Further adjusted for near visual acuity

### Stereopsis and cognitive impairment

Table 4 presents the association between stereopsis and cognitive impairment using a logistic regression analysis. Compared with the good stereopsis group, the moderate stereopsis group demonstrated a higher risk of cognitive impairment (OR = 1.72, 95% CI = 1.19, 2.49). After adjusting for all potential confounders, the moderate stereopsis group had a higher risk of cognitive impairment than the good stereopsis group (OR = 1.53, 95% CI = 1.03, 2.26). The poor stereopsis group also had a higher risk of cognitive impairment (OR = 2.13, 95% CI = 1.47, 3.08) than the good stereopsis group. The association remained even after including adjustment for all confounders (OR = 1.71, 95% CI = 1.14, 2.58).

**Table 4:** Logistic regression analyses to determine associations between stereopsis and cognitive impairment

	Model 1	Model 2	Model 3	Model 4	Model 5
	OR (95% CI)				
**Cognitive impairment**					
Good	*Ref.*	*Ref.*	*Ref.*	*Ref.*	*Ref.*
Moderate	**1.72 (1.19**, **2.49)**	**1.60 (1.10**, **2.34)**	**1.60 (1.09**, **2.33)**	**1.60 (1.09**, **2.34)**	**1.53 (1.03**, **2.26)**
Poor	**2.13 (1.47**, **3.08)**	**1.86 (1.27**, **2.73)**	**1.87 (1.28**, **2.75)**	**1.85 (1.26**, **2.73)**	**1.71 (1.14**, **2.58)**

Note: OR, odds ratio; CI, confidence interval. Model 1: Unadjusted; Model 2: Adjusted for age, sex, years of education, marital status, residence, and social security recipient; Model 3: Further adjusted for current smoking, alcohol intake, low physical activity, and body mass index; Model 4: Further adjusted for medical condition, and eye diseases (cataract, glaucoma, diabetic retinopathy, macular degeneration, and blindness); Model 5: Further adjusted for near visual acuity

## Discussion

To the best of our knowledge, this is the first study to demonstrate the association between stereopsis and cognitive function among community-dwelling older adults. Poor and moderate stereopsis was associated with higher odds of cognitive impairment, even after adjusted for eye diseases and visual acuity. Furthermore, poor stereopsis was negatively correlated with memory and executive function. These results provide evidence that poor stereopsis is associated with cognitive dysfunction and impairment.

Poor stereopsis was significantly associated with a higher risk of cognitive impairment after adjusting for confounding factors. This is consistent with findings from previous studies on patients with neurodegenerative diseases [37]. One cross-sectional study showed that cognitive dysfunction was associated with actual stereopsis perception using three-dimensional moving images among participants with Parkinson’s disease by using moving stimuli as a more advanced method for evaluating stereopsis [21]. Pathological findings explain the decline in visual function, including stereopsis, in patients with Parkinson’s disease, due to a lack of dopamine. The dopaminergic pathway also affects working memory and frontal executive function, which explains their close association [38]. A 2-year follow-up study of patients with Parkinson’s disease indicated that abnormal stereopsis is associated with rapid cognitive decline and the risk of developing dementia [37]. This finding is supported by a correlation between stereopsis and the non-dominant extrastriate visual cortex. In addition, visuospatial and perceptual deficits are associated with the development of dementia in patients with Parkinson’s disease [39]. Previous studies have shown that stereopsis deficits can lead to cognitive dysfunction in patients with neurodegenerative diseases. However, our study excluded participants with neurodegenerative diseases to ensure precise measurement of cognitive function. To accurately analyze the mechanisms of stereopsis and cognitive impairment, future studies should focus on patients without neurodegenerative diseases or other conditions that may affect cognitive function.

Our study showed that both poor and moderate stereopsis were associated with multiple cognitive function domains, including memory and executive function. In addition, poor stereopsis was significantly associated with attention and global cognitive function even after adjusting for all confounders. In a previous study that examined the relationship between visual function and cognitive decline, stereopsis demonstrated a cross-sectional association with executive function and a longitudinal relationship with language and memory [22]. Another finding of our study was that attention measured by the digit symbol test lost statistical significance in participants with moderate stereopsis after adjusting for visual acuity. In a longitudinal study, stereopsis impairment was not significantly associated with annual changes in digit symbol test scores, which assess executive function, although it was associated with a greater decline in composite cognitive function scores [36]. These results indicated that the number symbol test is a highly visual test that may present difficulties in assessing specific cognitive functions that are more susceptible to visual impairment.

Research examining stereopsis as an independent variable remains limited [22]; however, several studies have found the association between visual function and cognitive performance [40–42]. In a study of older Indian adults, Ehrlich et al. reported the association of vision impairment and cognitive performance in orientation, memory, and executive function domains [40]. Longitudinal studies have also demonstrated that poorer vision was associated with greater declines in language and memory, as well as global cognitive function, visuospatial organization, memory and verbal episodic memory [22, 42]. These findings implicate that visual impairment can cause diverse domain-specific effects on cognition. Though the reasons for these effects are unclear and limited to visual acuity among other visual function, our findings highlight the importance of assessing stereopsis to identify the underlying mechanisms by which visual impairment affects cognitive dysfunction in older adults.

Several studies have demonstrated the association between aging and decreased stereopsis. One explanation is that normal aging leads to a reduction in second-order stimulus processing, including stereopsis, which requires complex neural networks [43]. Another explanation is a reduction in cerebral function with aging [44]. The relationship between stereopsis and cognitive function can be elucidated by several hypotheses, given the number of factors involved in stereopsis [21]. Stereopsis, a key component of depth perception, is determined by the binocular disparity. A previous study demonstrated that binocular disparity, which is essential for depth perception, increases the cortical electrical activity in the frontal lobe and functional connectivity between the frontal and occipital lobes [45]. Our study also demonstrated a negative relationship between stereopsis and frontal lobe dysfunction. Because frontal lobe function has been used to define cognitive impairment, stereopsis deficits may contribute to frontal lobe dysfunction. A previous study has shown that participants with lower stereopsis have greater difficulty with inhibitory control, a type of executive function in community-dwelling older adults [46], which is in accordance with our result. We suggest that stereopsis is a significant predictor of cognitive impairment, given that reduced frontal lobe function leads to cognitive impairment [47]. Another theory is that vascular disease, a common cause of sensory and cognitive impairments, can cause both poor stereopsis and cognitive dysfunction [48]. A study of patients with branch retinal vein occlusion, a common vascular condition, showed improvements in stereopsis after injection treatment [48]. Our study only considered cardiovascular disease as a confounder, so further research that considers ocular vascular diseases is needed.

Our study has several strengths. First, this study used a neuropsychological battery test to assess the association between stereopsis and specific cognitive domains, whereas most previous studies have examined the association between vision and global cognitive function. Second, eye diseases (cataracts, glaucoma, diabetic retinopathy, macular degeneration, and blindness) were included as confounders. Third, we used the KFACS cohort data, which included a relatively large number of community-dwelling older adults who could fully represent the Korean older population. Despite its strengths, this study has some limitations. First, owing to its cross-sectional design, it was difficult to ascertain the causal relationship between stereopsis and cognitive impairment. Future longitudinal studies investigating the relationship between stereopsis and cognitive function are required. Second, contrast sensitivity is a significant factor in visual function along with visual acuity. A reduction in contrast sensitivity may negatively affect visual function. Therefore, we need to further clarify the relationship between visual function and cognitive dysfunction with a particular focus on contrast sensitivity. Third, although deficits in stereopsis are well-known to be associated with lesions in the occipitoparietal lobe, the relationship between these lesions and cognitive dysfunction remains unclear in community-dwelling older adults [19, 49]. Our study used FAB scores to evaluate the association between stereopsis and cognitive function, as frontal lobe function is a well-established measure of cognitive domains. Therefore, further research on the neural mechanisms linking the occipitoparietal areas and cognitive function is necessary to clarify visual-cognitive function in older adults. Fourth, since ocular pathologies were assessed based on medical history rather than clinical examination, inaccurate responses from patients may have influenced results.

## Conclusions

The results of this study indicate that, among community-dwelling older adults, poor stereopsis is related to cognitive dysfunction and a higher risk of cognitive impairment. Furthermore, memory and executive function are specific cognitive function domains that are significantly associated with poor and moderate stereopsis. Further longitudinal studies to confirm the causality between stereopsis deficits and cognitive function may provide novel insights for the management of cognitive impairment.

## Data Availability

The data used in the study is not publicly available, but the data used and/ or analyzed during the current study are available from the corresponding author on reasonable request. The datasets represented in this article are not readily available because of privacy and ethical restrictions. Requests to access the datasets should be directed to MK.

## Abbreviations

CERAD-K: Consortium to Establish a Registry for Alzheimer’s Disease Assessment Packet
LogMAR: Logarithm of the minimum angle of resolution
OR: Odds ratio
CI: Confidence Interval
MCI: Mild Cognitive Impairment
KFACS: Korean Frailty and Aging Cohort Study
MMSE: Mini-Mental State Examination
TMT-A: Trail Making Test A
FAB: Frontal Assessment Battery
BMI: Body Mass Index
